# A Role of Phosphatidylserine in the Function of Recycling Endosomes

**DOI:** 10.3389/fcell.2021.783857

**Published:** 2021-12-24

**Authors:** Junya Hasegawa, Yasunori Uchida, Kojiro Mukai, Shoken Lee, Tatsuyuki Matsudaira, Tomohiko Taguchi

**Affiliations:** Department of Health Chemistry, Graduate School of Pharmaceutical Sciences, University of Tokyo, Tokyo, Japan

**Keywords:** phosphatidylserine, pleckstrin-homology domain, flippase, bioID proximity labeling, endosomes, membrane traffic

## Abstract

Cells internalize proteins and lipids in the plasma membrane (PM) and solutes in the extracellular space by endocytosis. The removal of PM by endocytosis is constantly balanced by the replenishment of proteins and lipids to PM through recycling pathway. Recycling endosomes (REs) are specific subsets of endosomes. Besides the established role of REs in recycling pathway, recent studies have revealed unanticipated roles of REs in membrane traffic and cell signalling. In this review, we highlight these emerging issues, with a particular focus on phosphatidylserine (PS), a phospholipid that is highly enriched in the cytosolic leaflet of RE membranes. We also discuss the pathogenesis of Hermansky Pudlak syndrome type 2 (HPS2) that arises from mutations in the AP3B1 gene, from the point of view of dysregulated RE functions.

## Introduction

**FIGURE 1:**
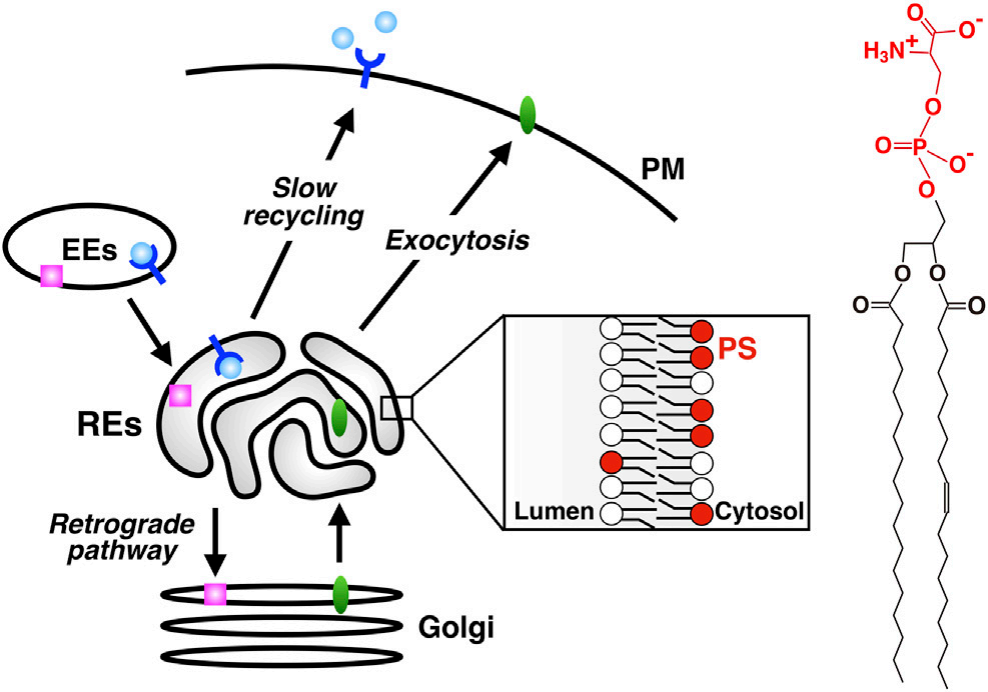
PS is enriched in REs, an organelle that serves as a hub for a variety of membrane traffic. slow recycling: PM→ EEs→ REs→ PM; retrograde transport: PM→ EEs→ REs→ the Golgi; exocytic transport: ER→ the Golgi→ REs→ PM. PS is concentrated in the cytosolic leaflet of RE membranes. Chemical structure of PS is shown in the right. The headgroup of PS (phosphoserine, shown in red) has one net negative charge.

Cells internalize proteins and lipids in the PM and solutes in the extracellular space by endocytosis. Internalized cargos are first transported to early endosomes (EEs). Cargos are further transported either to lysosomes through late endosomes for their degradation, or to the PM for their reuse. A direct route from EEs to the PM (the fast recycling pathway) and an indirect route through REs (the slow recycling pathway) are involved in the latter transport (Sheff et al., 1999; Sönnichsen et al., 2000). Alternatively, cargos can be transported to the Golgi by retrograde pathway (Bonifacino and Rojas, 2006; Johannes and Popoff, 2008). Some cargos bound to retrograde pathway pass through REs before reaching the Golgi (Mallet and Maxfield, 1999; Uchida et al., 2011; McKenzie et al., 2012). Furthermore, there is accumulating evidence that some exocytic cargos pass through REs before reaching the PM (Ang et al., 2004; Murray et al., 2005; Misaki et al., 2010). Thus, the classical view of REs, *i.e*., the organelle for recycling traffic, has been challenged and revised to the one that REs function as a hub for a variety of membrane traffic (Taguchi, 2013) (Figure 1).

PS represents up to 10% of the total phospholipids in cells and is the most abundant negatively charged glycerophospholipids (Leventis and Grinstein, 2010; Kay and Fairn, 2019). PS has a phosphoserine headgroup attached to the *sn*-3 position of the glycerol backbone. In mammals, PS is synthesized by two distinct base-exchange enzymes, PS synthase-1 (PSS1) and PS synthase-2 (PSS2). PSS1 substitutes serine for choline of phosphatidylcholine, whereas PSS2 replaces ethanolamine of phosphatidylethanolamine for serine (Kuge and Nishijima, 1997; Vance, 2018). These enzymes localize in the mitochondria-associated membranes of the ER (Vance, 2018). PS is highly enriched in the cytosolic leaflet of the PM and participates in various physiological events such as the coagulation cascade, recruitment and activation of signalling molecules that include protein kinase C, and clearance of apoptotic cells (Leventis and Grinstein, 2010). PS is also found in the cytosolic leaflet of intracellular organelles including EEs and late endosomes (Yeung et al., 2008), where its function has not been fully elucidated. Both vesicular membrane trafficking and non-vesicular transport by lipid transfer proteins appear to contribute to maintaining the subcellular PS distribution (Kay and Fairn, 2019).

Nearly a decade ago, we revealed that REs were enriched in PS. The finding was followed by a series of studies that identified the PS-specific protein domain, PS-effector RE proteins, and unanticipated roles of PS in the Hippo-YAP signalling. In this review, we summarize the role of PS in RE functions and discuss the pathogenesis of Hermansky Pudlak syndrome type 2 (HPS2) from the point of view of dysregulated PS/RE functions.

## Evectin-2, a PS Binding RE Protein

Evectin-1 and -2 were identified as post-Golgi proteins of unknown function (Krappa et al., 1999). Evectin-1 is expressed specifically in the nervous system, whereas evectin-2 is ubiquitously expressed. Both proteins are predicted to have a type IV membrane topology, *i.e*., the *N*-terminal part of the protein, which is anchored to the membrane by a *C*-terminal transmembrane domain, is oriented towards the cytosol (Krappa et al., 1999). They have a pleckstrin-homology (PH) domain at the *N*-terminus, which typically binds phosphoinositides (PIPs) (Lemmon, 2008). We showed that evectin-2 localized to REs and that evectin-2 PH was required for the localization of evectin-2 to REs (Uchida et al., 2011). Evectin-2 PH alone, expressed in the cytosol, localized to REs, indicating the presence of an RE-specific phospholipid.

**FIGURE 2:**
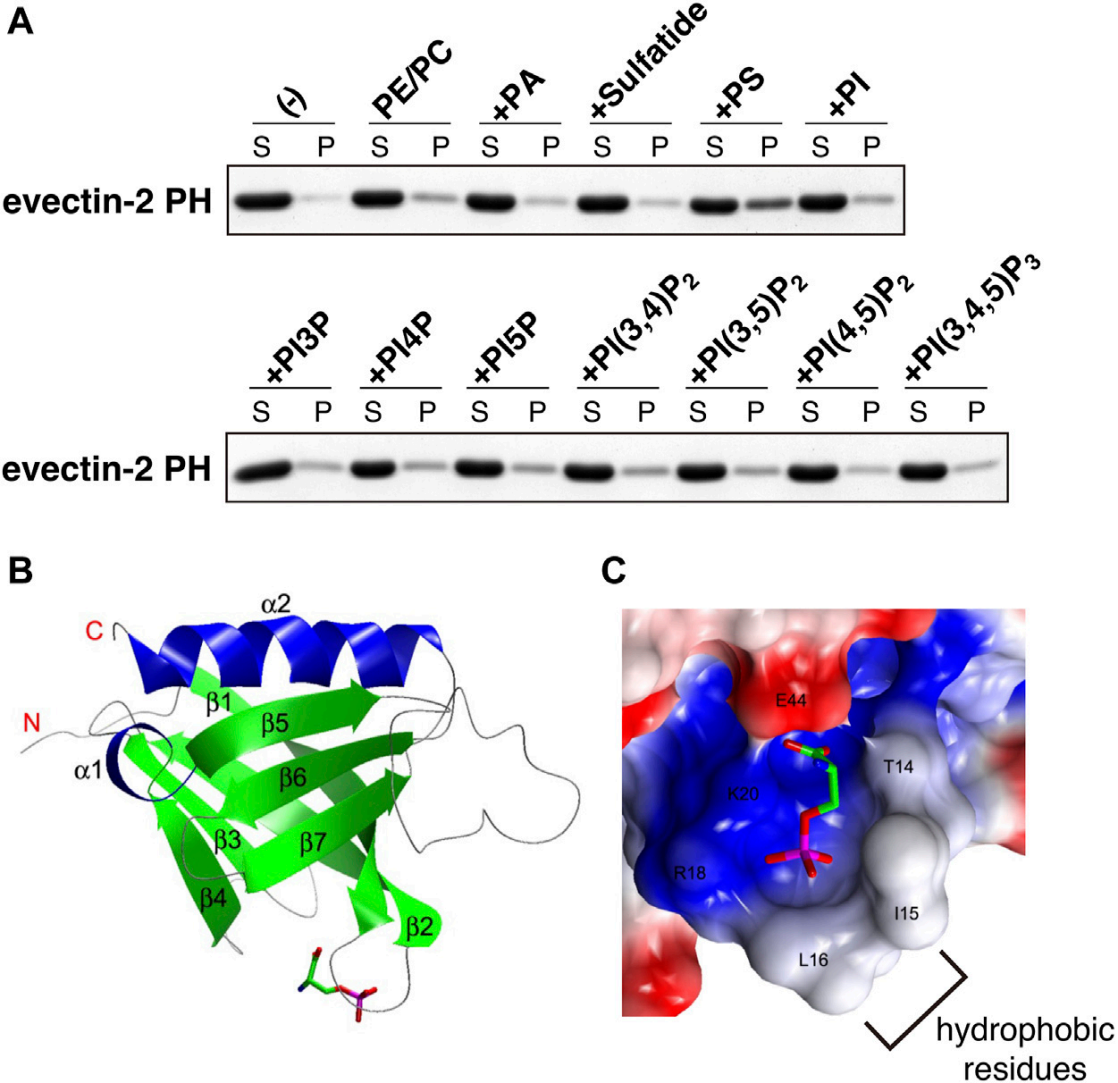
evectin-2 PH specifically binds to PS **(A)** *In vitro* lipid-binding assays. His-tagged evectin-2 PH was mixed with liposomes composed of brain phosphatidylcholine (PC), brain phosphatidylethanolamine (PE), and the indicated negatively charged lipid (20% mol/mol of total lipids). The mixture was then spun at 100,000 *g*, and the resultant supernatant (S) and pellet (P) were subjected to SDS-PAGE, followed by Coomassie blue staining **(B)** Overall structure of human evectin-2 PH in complex with phosphoserine, the headgroup of PS **(C)** Charge distribution surface model of evectin-2 PH in complex with phosphoserine (stick model). The surface is colored according to the electrostatic potential of the residues (blue, positive; red, negative). Hydrophobic residues around the PS-binding pocket, which are expected to be inserted into the membrane, are indicated. Data were reproduced and modified from (Uchida et al., 2011).

The human proteome has about 300 proteins with PH domains. About 10% of these proteins were shown to bind specifically to PIPs through their PH domains (Lemmon, 2008). A number of PH domains did not bind lipids but protein partners (Wang et al., 1994; Scheffzek and Welti, 2012). We measured the binding of several negatively charged lipids on liposomes to recombinant evectin-2 PH. Contrary to what we expected, PS bound evectin-2 PH, but phosphatidic acid, phosphatidylinositol, sulfatide, and all PIPs did not (Uchida et al., 2011) (Figure 2A). Lys20 is highly conserved in other PH domains. evectin-2 PH (K20E) lost the ability to bind to PS.

A *Saccharomyces cerevisiae* mutant (*cho1Δ*) lacks *de novo* PS synthesis and is devoid of PS (Atkinson et al., 1980; Hikiji et al., 1988). We exploited this yeast strain to examine if evectin-2 PH bound PS *in vivo*. Given that a tandem fusion of lipid-binding domains, such as the FYVE domain of EEA1 and Hrs, increased the lipid-binding affinity of their FYVE domain (Gillooly et al., 2000), a tandem fusion of evectin-2 PH (2xPH, hereafter) was generated. 2xPH localized exclusively at the PM of the wild-type yeast, whereas it was cytosolic in *cho1Δ*, indicating that evectin-2 PH recognized PS *in vivo* (Uchida et al., 2011). These results also indicated that 1) the cytosolic leaflet of RE membranes was enriched in PS and 2) evectin-2 localized to REs by the recognition of the PS at REs with its PH domain.

## Evectin-2 PH Domain as a PS Probe

**FIGURE 3:**
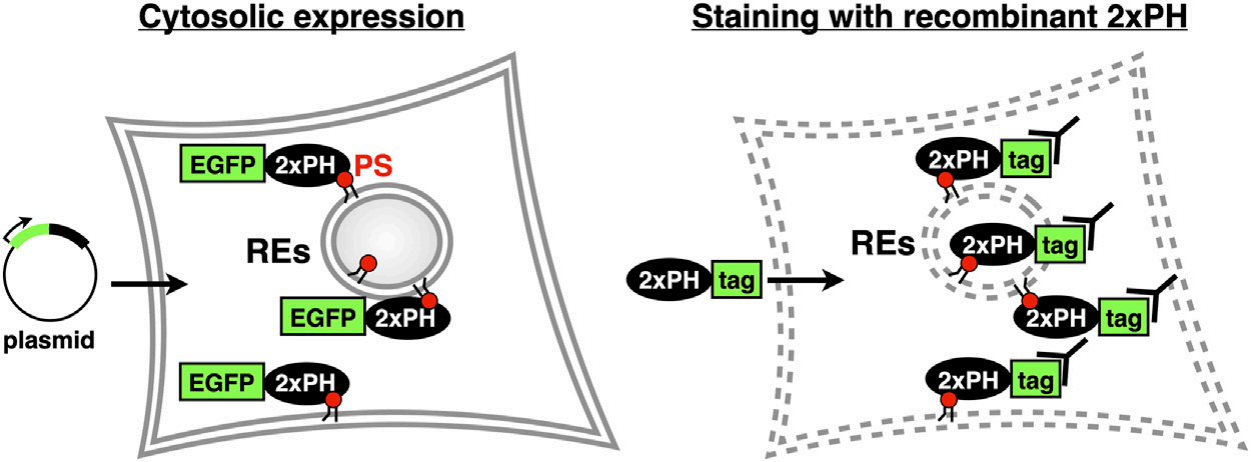
Two methods to examine intracellular PS distribution with 2xPH (*Left*) 2xPH tagged with a fluorescent protein, such as EGFP, is expressed in the cytosol by plasmid transfection. In this case, 2xPH detects PS in the cytosolic leaflet of the PM and organelle membranes (*Right*) Recombinant 2xPH detects PS in both leaflets when applied to fixed and permeabilized cells. 2xPH can be detected by immunocytochemistry using antibodies against the tag attached to 2xPH.

Since we reported that evectin-2 PH was highly specific to PS, this domain or the tandem fusion of evectin-2 PH (2xPH) has been widely used to examine the subcellular distribution of PS both in live and fixed cells (Table 1). If 2xPH tagged with a fluorescent protein, such as EGFP, is expressed in the cytosol, PS in the cytosolic leaflet of the PM and organelle membranes can be detected in live cells. If the recombinant 2xPH is used for fixed and permeabilized cells, PS in membranes, regardless of its transbilayer distribution, can be detected (Figure 3).

**TABLE 1:** Representative studies using 2xPH.

Model system	Purpose of using 2xPH	References
Mammalian cell line	Protein localization to PS-rich membranes	Chiba et al. (2013)
Mammalian cell line	PS transport to the PM	Chung et al. (2015)
Yeast	Change of PS distribution in genetic mutants	Hatakeyama et al. (2017)
Mammalian cell line	Identification of proteins in close proximity to PS	Matsudaira et al. (2017)
Plant	PS distribution in endosomal membrane	Platre (2018)
Plant	PS accumulation in nanodomains at the PM	Platre et al. (2019)
Yeast, mammalian cell line	Transbilayer PS distribution in organelle membranes	Tsuji et al. (2019)
Yeast	Change of PS distribution in genetic mutants	Kishimoto et al. (2021)
Mammalian cell line	PS levels in cellular membranes	Li et al. (2021)
Yeast	PS distribution in autophagosomes/autophagic bodies	Orii et al. (2021)

C2-domain of lactadherin (lact-C2, hereafter) has also been used to detect PS in cells (Yeung et al., 2008; Kay and Grinstein, 2011). Two papers used 2xPH and lact-C2 in the same cellular system: Platre et al. (Platre et al., 2018) found that the PM localization of lact-C2 was more pronounced than that of 2xPH; Chung et al. (Chung et al., 2015) reported that (total internal reflection fluorescence)/(epi fluorescence) with 2xPH was 0.1, whereas that with lact-C2 was 0.4. These results suggested that lact-C2 appeared more sensitive to detect PS in the PM than 2xPH. Intriguingly, Wen et al. (Wen et al., 2016) reported that 2xPH bound preferentially to PS in the liquid-disordered (Ld) phase, compared to PS in the liquid-ordered (Lo) phase using liposome reconstitution system. 2xPH, thus, may be susceptible to the lipid environment where PS is placed.

The crystal structure of evectin-2 PH with phosphoserine, the head group of PS, was solved (Uchida et al., 2011). By comparing the crystal structure of the apo-form of evectin-2 PH (Okazaki et al., 2012), Ile15 and Leu16 were found to be positioned closer to the PS-binding pocket upon PS binding (Figures 2B,C). The insertion of the hydrophobic side chains of Ile15 and Leu16 into densely packed lipid domains is expected to be energetically disfavored, which may account for the *in vitro* 2xPH preference for PS in the Ld phase rather than PS in the Lo phase.

## ATP8A1, an RE-Localized PS Flippase

**FIGURE 4:**
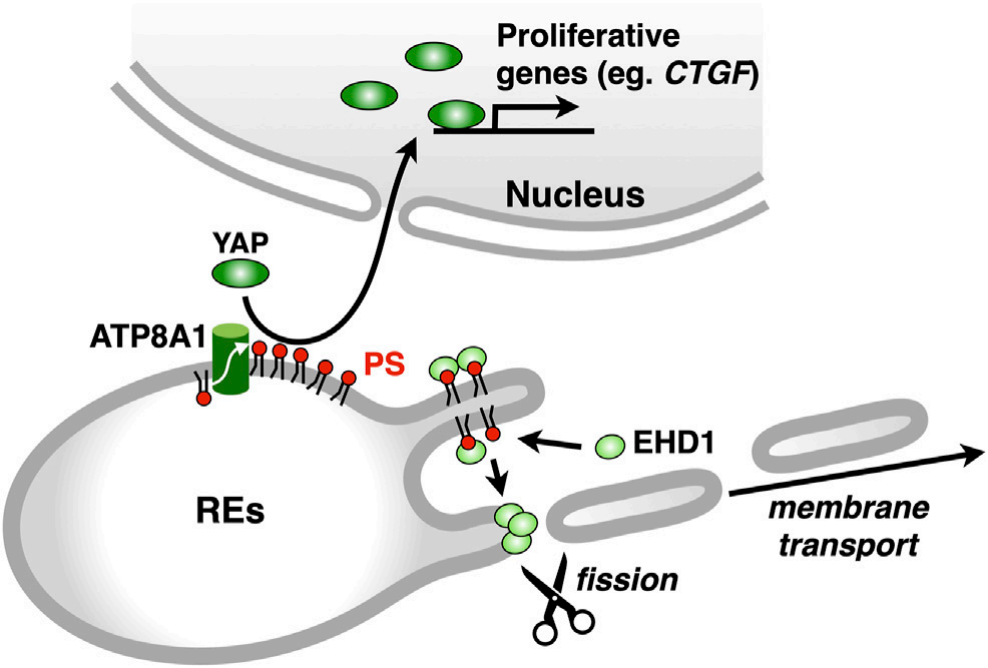
ATP8A1 regulates membrane trafficking and signalling at REs. ATP8A1 flips PS to the cytosolic leaflet of RE membranes. The PS then recruits a membrane fission protein EHD1 from the cytosol to REs. The EHD1-mediated fission of RE membranes generates membrane transport carriers. PS in REs also facilitates the nuclear translocation of YAP, thereby promoting the transcription of YAP-target proliferative genes, such as CTGF (connective tissue growth factor).

Asymmetric distribution of phospholipids in the lipid bilayer is generated, in part, by the selective translocation of phospholipids across the membranes (Graham, 2004; Holthuis and Levine, 2005; Best et al., 2019). The P_4_ subfamily of P-type ATPases (P_4_-ATPases) flips phospholipids from the luminal leaflet (or extracellular leaflet) to the cytosolic leaflet of biomembranes (Sebastian et al., 2012; Coleman et al., 2013). Fourteen P_4_-ATPases are encoded in human genome, and mutations in some P_4_-ATPases cause genetic diseases, such as intrahepatic cholestasis (Bull et al., 1998), B-cell deficiency syndrome (Siggs et al., 2011; Yabas et al., 2011), and neurodegenerative disorder (Onat et al., 2013). We sought to identify the P_4_-ATPase that concentrates PS in the cytosolic leaflet of RE membranes. Four P_4_-ATPases (ATP8A1, ATP9A, ATP11A, and ATP11B) are ubiquitously expressed and suggested to localize at endosomes (Takatsu et al., 2011; Kato et al., 2013). We found that ATP8A1 localized at REs and its knockdown resulted in an increase in PS levels in the luminal leaflet of RE membranes (Lee et al., 2015). Knockdown of ATP8A1 also impaired the recycling of transferrin (Tfn) and the retrograde traffic of cholera toxin B subunit (CTxB) at REs. Importantly, the rescue experiments with siRNA-resistant ATP8A1 E191Q (an ATPase-deficient variant) showed that ATPase activity of ATP8A1 was required for the recycling of Tfn from REs. These results suggested that PS in the cytosolic leaflet of RE membranes was essential for membrane traffic that passes through REs. Intriguingly, knockdown of ATP8A1 resulted in the generation of aberrant tubules that were positive with Tfn receptor (TfnR). PS in the cytosolic leaflet of RE membranes may function in the fission process of membrane carriers (Figure 4).

The function of PS flippases in endosomal membrane traffic appears to be evolutionally conserved. For example, a P_4_-ATPase Drs2 in *Saccharomyces cerevisiae*, which flips PS, is essential for membrane traffic between the late Golgi compartment and endosomes (Best et al., 2019). Drs2 increases membrane curvature and anionic phospholipid levels by providing an excess of lipids in the cytosolic leaflet of the membrane, both of which are sensed by the Arf GTPase-activating protein (ArfGAP) Gcs1 through its +ALPS motif (Xu et al., 2013). By analogy, ATP8A1 may also contribute to membrane traffic through REs by creating positive membrane curvature, which is essential for generating membrane carriers. A P_4_-ATPase TAT-1 in *Caenorhabditis elegans*, is most closely related to mammalian ATP8A1. The loss of *tat-1* leads to the generation of abnormal endo-lysosomal compartments, suggesting impaired endocytic traffic (Ruaud et al., 2009; Chen et al., 2010). Of note, ATP9A, another P4-ATPase that localizes at endosomes, is required for the efficient recycling of Tfn from endosomes to the PM (Tanaka et al., 2016). It is currently unclear whether ATP9A is involved in the enrichment of PS in the cytosolic leaflet of endosomal membranes.

ATP8A2, a paralogue of ATP8A1, is specifically expressed in brain, testis, and retina (Zhu et al., 2012). An ATP8A2 variant (I376M) is associated with a neurodegenerative disease (CAMRQ) characterized by cerebellar ataxia, mental retardation, and disequilibrium (Onat et al., 2013). We hypothesized that ATP8A2, like ATP8A1, was essential for endosomal traffic through REs. Thus, three human ATP8A2 variants [wild-type (WT), I376M, and E210Q deficient in flippase-activity] were examined if the expression of these could compensate for the loss of ATP8A1. All three ATP8A2 proteins localized at REs, however, only the expression of ATP8A2 (WT), in cells depleted of ATP8A1, resulted in the disappearance of aberrant TfnR-positive tubules and restored EHD1 localization to REs (Lee et al., 2015) (please see the following section). These results suggested that ATP8A2 functioned in recycling endosomal traffic. The defect in recycling endosomal traffic in neurons may underlie the pathogenesis of CAMRQ.

## EHD1, a PS-Effector RE Protein

Eps15 homology domain-containing protein 1 (EHD1) is a member of the EHD (EH-domain containing) family, which contains four homologues in mammals designated EHD1, EHD2, EHD3, and EHD4. These proteins are highly conserved eukaryotic dynamin-like ATPases that mediate membrane remodeling (Daumke et al., 2007; Grant and Caplan, 2008). EHD1 facilitates tubulation or fission of liposomes containing anionic phospholipids, suggesting that EHD1 functions in the formation of membrane carriers *in vivo* (Pant et al., 2009). Knockdown of EHD1 impaired the recycling of Tfn from REs to the PM and the retrograde transport of CTxB from REs to the Golgi (McKenzie et al., 2012; Lee et al., 2015). Intriguingly, EHD1 knockdown, like ATP8A1 knockdown, resulted in the emergence of aberrant TfnR-positive tubules emanating from REs.

Given the similar phenotype of the distribution of TfnR between ATP8A1-and EHD1-depleted cells, we hypothesized that PS in the cytosolic leaflet of RE membranes regulated EHD1 function. Indeed, we found that EHD1 localized primarily at REs in WT cells, but not in ATP8A1-depleted cells. Furthermore, the RE localization of EHD1 in ATP8A1-depleted cells was restored by the expression of siRNA-resistant WT ATP8A1, but not by the expression of siRNA-resistant E191Q mutant deficient in ATPase activity (Lee et al., 2015). Co-sedimentation assays with recombinant EHD1 mixed with liposomes of increasing PS levels showed a sigmoidal increase in the EHD1 binding, with an EC50 of 40–50 mol% PS. This PS concentration matched well the concentration of PS in the cytosolic leaflet of RE membranes estimated with a method using recombinant 2xPH (Lee et al., 2015). Thus, PS in the cytosolic leaflet of RE membranes by itself may recruit EHD1 from the cytosol, thereby facilitating its function to generate membrane carriers for the PM and/or the Golgi (Figure 4).

## Regulation of the YAP Signalling by PS in REs

Given the presence of RE proteins, such as evectin-2 and EHD1, the localization of which depends on the levels of PS in the cytosolic leaflet of RE membranes, we hypothesized that there were more PS-binding RE proteins. To identify these proteins, the proximity-dependent biotin identification (BioID) method was exploited. The BioID method is based on proximity-dependent cellular biotinylation by a promiscuous bacterial biotin ligase BirA* fused to a bait protein (Roux et al., 2012). Biotinylated proteins can be purified by avidin-coated beads, and subsequently identified using mass spectrometry analysis.

As the bait protein, we used 2xPH, expecting that RE proteins in close proximity to PS could be biotinylated. Among 400 biotinylated proteins identified, 113 proteins were annotated to “endosomes” in gene ontology in Uniprot. Several proteins that function in membrane trafficking at REs were identified, including EHD1, VAMP3 (McMahon et al., 1993), Rab11-FIP1 (Hales et al., 2001), MICAL-L1 (Sharma et al., 2009), and SMAP2 (Matsudaira et al., 2013; Matsudaira et al., 2015). Intriguingly, we found that YAP, a critical growth-promoting transcription coactivator, and a group of proteins associated with the YAP signalling pathway (the Hippo pathway) were biotinylated with BirA*-2xPH (Matsudaira et al., 2017). These results suggested that PS in the RE membranes was involved in the YAP signalling. Indeed, we found that YAP localized at REs in low-density proliferating cells, in addition to its expected localization of the nucleus, where YAP regulates target genes that are essential for cell proliferation (Zhao et al., 2007; Zhao et al., 2008). Knockdown of ATP8A1 reduced the nuclear/RE localization of YAP and the mRNA expression of CTGF, a YAP-regulated gene (Matsudaira et al., 2017). These results suggested that PS in REs had a role in the YAP activation (Figure 4). Whether YAP directly binds to PS at REs remains to be elucidated. Knockdown of evectin-2 also reduced the nuclear/RE localization of YAP and the mRNA expression of CTGF. The regulation of YAP by evectin-2 was suggested to be mediated through the direct activation of Nedd4 E3 ligases, such as Itch, WWP1, and WWP2, by evectin-2. These E3 ligases ubiquitinated Lats1 kinase, the critical negative regulator of YAP function, leading to proteasome degradation of Lats1.

## Hermansky Pudlak Syndrome Type 2

Hermansky-Pudlak syndrome (HPS) is a rare, hereditary disorder characterized by decreased pigmentation (albinism) with visual impairment, blood platelet dysfunction with prolonged bleeding, and pulmonary fibrosis (Vicary et al., 2016; Bowman et al., 2019). The most lethal complication in HPS patients is pulmonary fibrosis. So far, human genetics identify more than 10 separate forms of HPS with mutations in different genes (Vicary et al., 2016). All HPS have defect in membrane trafficking and the biogenesis of lysosome-related organelles (LROs), including melanosomes and platelet dense granules (Bowman et al., 2019). Hermansky Pudlak syndrome type 2 (HPS2) is caused by mutations in AP3B1 gene, which encodes β1 subunit of the adaptor protein 3 (AP-3) complex (Dell’Angelica et al., 1999). AP-3 serves as a protein coat of membrane vesicles and mediates the transport of transmembrane proteins to lysosomes or LROs (Bowman et al., 2019). Although dysregulation of alveolar epithelial cells appears critical to the pathogenesis of HSP, the molecular mechanism by which the fibrosis proceeds is largely unknown.

**FIGURE 5:**
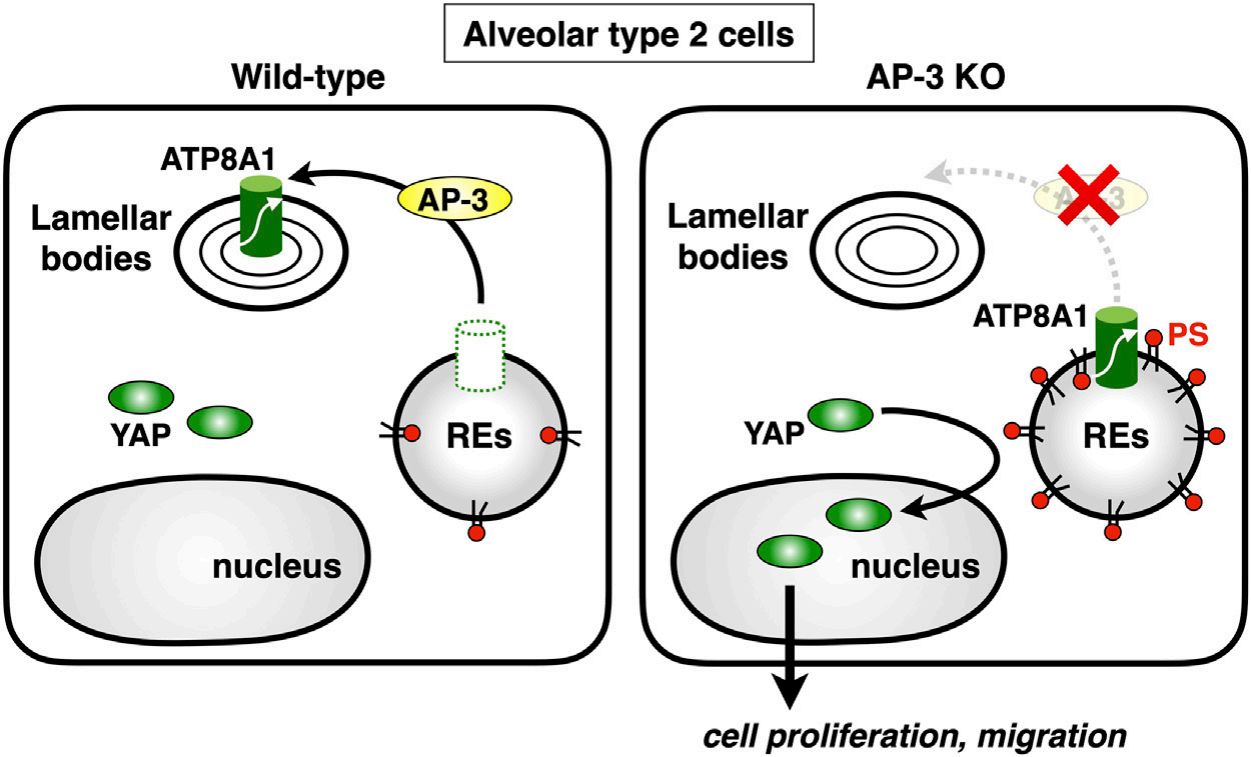
Deficiency of AP-3 results in YAP activation. AP-3 mediates ATP8A1 transport from endosomes to lamella bodies in alveolar type 2 cells. In AP-3-knockout cells, because the trafficking of ATP8A1 from endosomes is impaired, ATP8A1 is forced to re-localize to REs, thereby increasing the levels of cytosolic PS in RE membranes. The PS enrichment in RE membranes promotes aberrant activation of YAP, which augments cell proliferation and migration.

Lamellar bodies (LBs) are LROs of surfactant-producing alveolar type 2 (AT2) cells of the distal lung epithelium (Weaver et al., 2002). A recent study showed that ATP8A1 in AT2 cells was constantly transported to LBs by AP-3 (Kook et al., 2021). Interestingly, instead of being transported to LBs, ATP8A1 in AP-3-depleted cells re-localized to REs, enhancing the cytosolic exposure of PS in REs, as we reported in other cell lines (Lee et al., 2015). This, in turn, promoted activation of YAP, enhancing cell migration and AT2 cell numbers (Kook et al., 2021) (Figure 5). Thus, the dysregulated PS exposure in REs may in part contribute to the pathogenesis of HSP2.

## Concluding Remarks

Nearly a decade ago when we investigated the mechanism by which evectin-2 localized at REs, we serendipitously found that the cytosolic leaflet of RE membranes were enriched in PS (Uchida et al., 2011). The enrichment of PS at the cytosolic leaflet of RE membranes highly contrasts with the specific expression of PIPs at the cytosolic leaflet of other membrane compartments, *e.g*., PI(3)P at EEs and PI(4,5)P_2_ at the PM (Schink et al., 2016). Given a variety of PIP effectors that regulate organelle function (Di Paolo and De Camilli, 2006), we reason that more PS effectors, in addition to evectin-2 and EHD1, exist and contribute to the function of REs. The BioID methods should help identify these.

Besides the classical roles of REs in endocytic recycling, we and others have shown that REs have a role in the exocytic and retrograde membrane traffic. These results raise a fundamental question how individual cargos are packaged into appropriate membrane carriers. *In vivo* imaging system to visualize the dynamics of multiple cargos for distinct destinations, and *in vitro* reconstitution system, such as those developed for the early secretory pathway (Kim et al., 2005; Kim et al., 2007), would greatly benefit in our understanding of the nature and regulators of membrane traffic at REs.
